# Genome-wide characterization of the *MBF1* gene family and its expression pattern in different tissues and stresses in *Zanthoxylum armatum*

**DOI:** 10.1186/s12864-022-08863-4

**Published:** 2022-09-14

**Authors:** Wenkai Hui, Hao Zheng, Jiangtao Fan, Jingyan Wang, Tahseen Saba, Kai Wang, Jiaojiao Wu, Han Wu, Yu Zhong, Gang Chen, Wei Gong

**Affiliations:** Key Laboratory of Ecological Forestry Engineering of Sichuan Province, College of Forestry, Sichuan Agricultural University, Chengdu, 611130 China

**Keywords:** *Zanthoxylum armatum*, MBF1, Stress response, Expression pattern

## Abstract

**Background:**

Multiprotein bridging factor 1 (MBF1) is a crucial transcriptional coactivator in animals, plants, and some microorganisms, that plays a necessary role in growth development and stress tolerance. *Zanthoxylum armatum* is an important perennial plant for the condiments and pharmaceutical industries, whereas the potential information in the genes related to stress resistance remains poorly understood in *Z*. *armatum*.

**Results:**

Herein, six representative species were selected for use in a genome-wide investigation of the *MBF1* family, including *Arabidopsis thaliana*, *Oryza sativa*, *Populus trichocarpa*, *Citrus sinensis*, *Ginkgo biloba*, and *Z. armatum*. The results showed that the *MBF1* genes could be divided into two groups: Group I contained the MBF1a and MBF1b subfamilies, and group II was independent of the MBF1c subfamily.. Most species have at least two different *MBF1* genes, and *MBF1c* is usually an essential member. The three *ZaMBF1* genes were respectively located on ZaChr26, ZaChr32, and ZaChr4 of *Zanthoxylum* chromosomes. The collinearity were occurred between three *ZaMBF1* genes, and *ZaMBF1c* showed the collinearity between *Z. armatum* and both *P. trichocarpa* and *C. sinensis*. Moreover, many cis-elements associated with abiotic stress and phytohormone pathways were detected in the promoter regions of *MBF1* of six representative species. The ERF binding sites were the most abundant targets in the sequences of the *ZaMBF1* family, and some transcription factor sites related to floral differentiation were also identified in *ZaMBF1c*, such as *MADS*, *LFY*, *Dof*, and *AP2*. *ZaMBF1a* was observed to be very highly expressed in 25 different samples except in the seeds, and *ZaMBF1c* may be associated with the male and female floral initiation processes. In addition, expression in all the *ZaMBF1* genes could be significantly induced by water-logging, cold stress, ethephon, methyl jasmonate, and salicylic acid treatments, especially in *ZaMBF1c*.

**Conclusion:**

The present study carried out a comprehensive bioinformatic investigation related to the MBF1 family in six representative species, and the responsiveness of *ZaMBF1* genes to various abiotic stresses and phytohormone inductions was also revealed. This work not only lays a solid foundation to uncover the biological roles of the *ZaMBF1* family in *Z*. *armatum*, but also provides some broad references for conducting the *MBF1* research in other plants.

**Supplementary Information:**

The online version contains supplementary material available at 10.1186/s12864-022-08863-4.

## Background

Flooding, drought, salinity, metal ions, extreme temperature and other adverse conditions severely inhibit plant growth and development, often resulting in changes in plant physiology, biochemistry, morphology, senescence and even death [1]. Therefore, carrying out studies on plant stress and improving plant resistance can improve the economic values of the plant. The vegetative growth, reproduction, and development of plants are significantly impacted by a vast array of biotic and abiotic stresses [2]. During long-term evolution, plants have developed a comprehensive regulatory network to facilitate the rapid detection of environmental changes and simultaneously conduct adaptive responses [3]. Environmental signals can be sensed by internal stress-induced factors in plants, and then a broad range of transcription factors (TFs) will be activated to mediate the plant appropriate responses, including WAKY, Trihelix, NAC, MYB, ERF, C2H2, bZIP, bHLH, LBD, MBF1, and other families [1, 4–6]. In previous studies, approximately 7% of the plant genomecould be encoded TFs, including approximately 4.7% of *Arabidopsis thaliana*, 6.4% of poplar, and 7.7% of *Zanthoxylum armatum* [2, 7, 8]. These TFs, as signal molecules, interact with specific cis-acting elements in the promoter region of downstream stress response genes to regulate the signal cascade network in plants [8–10].

Multiprotein bridging factor 1 (MBF1), as one of the crucial transcriptional coactivators, is widely distributed in animals, plants, and some microorganisms, and plays a necessary role in gene expression and regulation during stress responses [11, 12]. There are three members in the MBF1 family, including MBF1a, MBF1b, and MBF1c [13], each with a flexible N-terminal region and highly conserved helix-turn-helix (HTH) domain in its C-terminal [11]. The function of the N-terminus was to bind with the activating proteins, and the C-terminus was contributed to the protein folding and dimer formation of functional MBF1. In previous studies, MBF1, as a transcriptional coactivator, has been shown to bridge the regulatory factor and TATA-box binding protein. It continually shifts its the locations in the cytoplasm and nucleus to conduct its functions [14]. The ubiquitous expression of *MBF1*s was investigated in various tissues of different species, such as roots, stems, leaves, flowers, fruits, seeds and anther organs [15], but some specific expression abundances were found among the three different *MBF1* genes during plant development [13, 16]. The overexpression of *AtMBF1c* can increase the number of seeds in *Arabidopsis* and soybeans [17, 18], and the flowering time was also earlier in *Arabidopsis* [17]. MBF1c rapidly accumulates and interacts with trehalose-phosphate synthases to respond to heat stress [19]. The overexpression of *MBF1* genes also increases antioxidant enzyme activity under drought stress and NaCl treatment [20, 21]. A subsequent study found that an increase in the Ca^2+^ signal could enhance tolerance to salt stress in *MBF1c* overexpressing plants [22]. *MBF1* genes appear to be the negative regulator for cold stress response in hot pepper [15], but have no effect on seedling in *Arabidopsis* and *R. raetam* [15]. Although some previous reports described the MBF1 genes in other species, there is a paucity of information at the whole genome level. Therefore, more functions of MBF1 need to be continuously uncovered in plants.

*Zanthoxylum* (Rutaceae) is one of the most important economic and medicinal tree species in southwestern China and some regions of Asia, such as Japan, South Korea, India, Pakistan, Nepal and so on [23, 24]. Due to the special numbing taste of its fruits, it is a very famous and essential ingredient in Sichuan hot-pot in China, and is often used to flavour some delicious dishes in many countries [25, 26]. *Zanthoxylum armatum* DC., commonly named green prickly ash, is not only enriched in special numbing substances, but also has a special fragrance, and the colour of its fruit is green, all of which contribute to its increasing popularity in recent public diets [27]. As one of the crucial spices to these cuisines and regimens, this agricultural product creates commercial value in billions of dollars in China, as shown on the website of huajiao.cn [24, 28]. Meanwhile, *Z. armatum* is also used for economic development as a vital medicinal plant in several countries [29–31]. For instance, *Z*. *armatum* is planted as the second most important medicinal species in Pakistan [32]. The fruit pulp of *Zanthoxylum* could be extended to treat skin diseases, toothache, fever, and stomach ache [24, 26, 30]. Plant-based medications are a high proportion of prescription used in regions of health care scarcity [33]. Fortunately, *Z*. *armatum* exhibits a high tolerance to drought, heat, and nutrient poor soils, so it is also selected to colonize barren mountains as a pioneer tree. However, *Z*. *armatum* is susceptible to flooding, cold, and some disease, which seriously restrict its large-scale application and development, especially in tropical and subtropical rainy regions. Therefore, exploring stress resistance is a more effective strategy to breed superior varieties and expand the cultivation regions of *Z*. *armatum*, thereby increasing the availability of its products and economic value. In a previous study, 38 WRKY transcription factors were identified in response to drought in *Zanthoxylum bungeanum* Maxim (red prickly ash) [25]. However, the potential genetic information of the genes related to stress resistance remains poorly understood in *Z*. *armatum* (green prickly ash).

Recently, the genomic dataset of *Z*. *armatum* was released with important resources [34], providing a wealth of information to explore the gene families associated with resistance functions. Thus, in the present study, the *MBF1* family was isolated and identified in *Z*. *armatum*. To comprehensively obtain the detailed characteristics of the *MBF1* family, we also selected another five species, including *Arabidopsis thaliana* (Herbage), *Oryza sativa* (Poaceae), *Populus trichocarpa* (Xylophyta), *Citrus* (Homology), and *Ginkgo biloba* (Gymnosperm). A series of analyses were performed in the present study to identify features, such as phylogenetic relationships, gene structures, motif compositions, chromosomal distributions, gene duplications, cis-acting elements in promoters, protein tertiary structures, and target gene sites. The gene expression profiles of the *ZaMBF1* family were also carried out in 26 different tissues by using publicly available and proprietary RNA-seq datasets. Furthermore, we also investigated the expression patterns of *ZaMBF1* genes in response to various abiotic stresses and phytohormone inductions by qRT-PCR analysis, including water-logging, cold stress, ethephon, methyl jasmonic acid (MeJA), and salicylic acid (SA) treatment. Thus, the results described in the present study represent valuable information for future functional studies associated with the MBF1 family.

## Results

### Isolation and identification of MBF1 in six species

To comprehensively analyse the information of the MBF1 family in the present study, six representative species were selected to isolate the MBF1 genes, including *A. thaliana* as the herbage model plant, *O. sativa* as the Poaceae model plant, *P. trichocarpa* as the woody model plant, *C. sinensis* as the homology plant, *G. biloba* as the gymnosperm plant, and *Z. armatum*. Fortunately, most of these species had three MBF1 genes, and there were five members in *C. sinensis* (Table 1). The TAIR11 annotation showed that three different subfamilies were identified in *O. sativa* and *Z. armatum,* named *MBF1a*, *MBF1b*, and *MBF1c*. Meanwhile, two *MBF1b* and one *MBF1c* members were identified in *P. trichocarpa*, whereas one *MBF1b* and two *MBF1c* members were identified in *G. biloba*. The lengths of the genomic DNA (gDNA) and coding sequence (CDS) were 222–23,512 bp and 222–468 bp, respectively. The CDS lengths of *MBF1c* were longer than those of *MBF1a* and *MBF1b* in various species, except for *C. sinensis*. It was interesting to note that most MBF1 proteins were observed to be approximately 140–155 amino acids, except for *OsMBF1b*, *CsMBF1b.1*, *CsMBF1b.3*, *CsMBF1b.4*, and *CsMBF1c*.

**Table 1:** The information summary of MBF1 sequences and annotation

Gene name	Gene ID	gDNA	CDS	GC %	AA	MW	pI	GRAVE	Pfam ID	Top Hit Description	TAIR BlastX
AtMBF1a	AT2G42680.1	1608	429	49.88	142	15.62	9.9	-0.78	PF08523.10/PF01381.22	MBF 1	/
AtMBF1b	AT3G58680.1	1864	429	49.42	142	15.58	9.99	-0.82	PF08523.10/PF01381.22	MBF1	/
AtMBF1c	AT3G24500.1	1180	447	46.98	148	16.4	9.99	-0.69	PF08523.10/PF01381.22	MBF1	/
OsMBF1a	LOC_Os08g27850.1	3230	429	57.58	142	15.63	9.95	-0.84	PF08523.10/PF01381.22	MBF1	AT2G42680.1
OsMBF1b	LOC_Os08g27850.2	839	354	58.47	117	12.77	10.22	-0.8	PF08523.10/PF01381.22	MBF1	AT3G58680.1
OsMBF1c	LOC_Os06g39240.1	3244	468	75	155	16.15	10.67	-0.4	PF08523.10/PF01381.22	MBF1	AT3G24500.1
PtMBF1b.1	Potri.001G390400.2	2524	429	50.82	142	15.46	9.91	-0.81	PF08523.10/PF01381.22	MBF1	AT3G58680.1
PtMBF1b.2	Potri.011G109500.1	2358	423	49.88	140	15.36	9.91	-0.78	PF08523.10/PF01381.22	MBF1	AT3G58680.1
PtMBF1c	Potri.018G075200.1	727	438	53.42	145	16.07	10.03	-0.71	PF08523.10/PF01381.22	MBF1	AT3G24500.1
CsMBF1b.1	Cs5g12510.1	2340	315	51.75	104	11.26	9.79	-0.77	PF08523.10	MBF1	AT3G58680.1
CsMBF1b.2	Cs5g12510.2	2340	429	50.35	142	15.57	9.91	-0.78	PF08523.10/PF01381.22	MBF1	AT3G58680.1
CsMBF1b.3	Cs5g12510.3	2340	381	49.34	126	13.85	9.56	-0.58	PF08523.10	MBF1	AT3G58680.1
CsMBF1b.4	Cs5g12510.4	2340	354	44.07	117	12.91	9.63	-0.33	PF01381.22/PF08523.10	Helix-turn-helix	AT3G58680.1
CsMBF1c	Cs4g16700.1	222	222	51.8	73	8.35	10.34	-0.94	PF01381.22/PF08523.10	Helix-turn-helix	AT3G24500.1
GbMBF1b	evm.model.chr1.2461	23,512	429	44.99	142	15.62	10.03	-0.73	PF08523.10/PF01381.22	MBF1	AT3G58680.1
GbMBF1c.1	evm.model.chr7.1473	435	435	52.64	144	15.95	10.24	-0.8	PF08523.10/PF01381.22	MBF1	AT3G24500.1
GbMBF1c.2	evm.model.chr7.1475	459	459	52.72	152	16.7	10.15	-0.76	PF08523.10/PF01381.22	MBF1	AT3G24500.1
ZaMBF1a	Zardc37022	1960	429	49.18	142	15.59	9.91	-0.79	PF08523.10/PF01381.22	MBF1	AT2G42680.1
ZaMBF1b	Zardc43131	1916	429	49.18	142	15.67	9.91	-0.82	PF08523.10/PF01381.22	MBF1	AT3G58680.1
ZaMBF1c	Zardc06390	608	453	53.86	150	16.59	10.15	-0.73	PF08523.10/PF01381.22	MBF1	AT3G24500.1

Note: Gene ID is the ID in their genome database, gDNA is the base length of DNA sequence, CDS is the base length of conding sequence, GC % is the content of G and C base in Coding sequence, AA is the number of amino acid, MW is the molecular weight (kDa), GRAVY is the grand average of hydropathicity, pI is the theoretical isoelectric point

To further clarify the characteristics of MBF1 family in *Z. armatum*, we analysed the protein molecular weight (MW), isoelectric point (pI), grand average of hydropathicity (GRAVY), and Pfam annotation. It was clearly found that all twenty MBF1 proteins exhibited overall hydrophilicity (from -0.94 to -0.33). The same Pfam IDs suggested that these proteins contained the same conserved domain (Additional file 3: Table S3). In general, the ZaMBF1 family has shown typical molecular characteristics compared with other different species, which will be helpful for further study to uncover its specific biological functions.

### Evolutionary and phylogenetic analysis of the MBF1 family

**Fig. 1:**
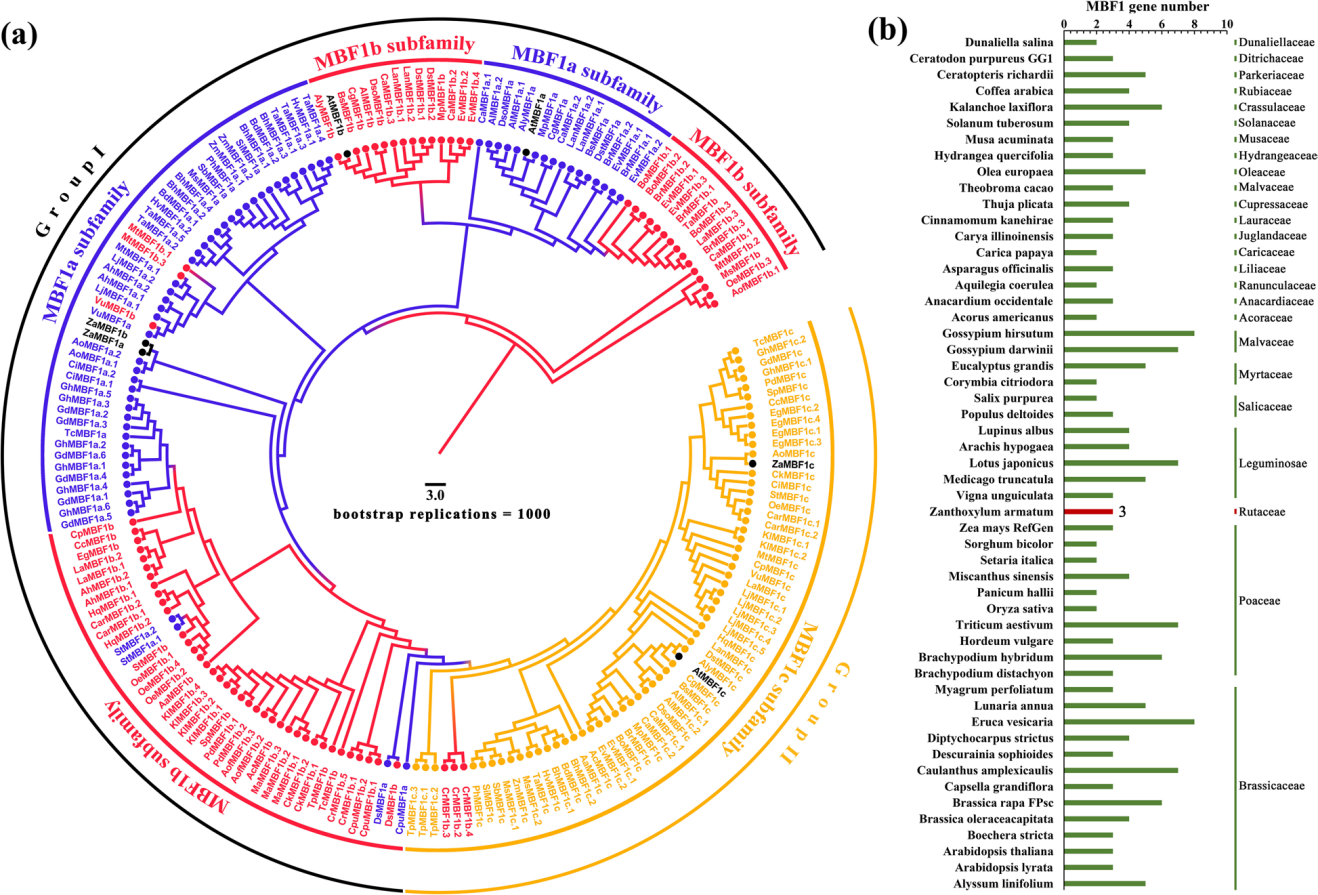
The phylogenetic analysis of MBF1 family in various plant species. **a** The phylogenetic tree of MBF1 proteins in 53 species. The different colors represent the three different MBF1 subfamilies, and the Z. armatum and A. thaliana were marked in dark fonts. **b** The summary and comparisons of MBF1 number among 53 plant species

In the present study, a total of 202 amino acid sequences from 53 species were collected to construct a phylogenetic analysis and illustrate the evolutionary relationships of MBF1 family proteins (Fig. 1a). The results showed that all of the MBF1s were divided into two groups based on their subfamilies. Group I contained the MBF1a and MBF1b subfamilies, and group II independently comprised the MBF1c subfamily. MBF1a and MBF1b were detected to have a closer relationship, which suggested that they might conduct more similar functions. However, MBF1c was displayed to be a relatively independent group, which indicated that it might play some specific roles related to stress response and the development process in plants. Moreover, in *Anacardium occidentale*, a member of the Anacardiaceae family, three MBF1 proteins were adjacent to the ZaMBF1 protein. Notably, both of ZaMBF1a and ZaMBF1b were located in the MBF1a subfamily. Combined with their similar sequence information (Table 1), we suggest that ZaMBF1a and ZaMBF1b might play similar roles or even redundant functions.

To explore the origin and evolution of *MBF1* genes, we investigated 53 species from different kinds of plants. Notably, the number of *MBF1* genes ranged from 2 to 8, and most plants contained 3 or 4 genes (Fig. 1b). More *MBF1* genes were detected in annual or perennial herbs (Additional file 1: Table S1), such as *Brassica rapa* FPsc (6), *Caulanthus amplexicaulis* (7), *Eruca vesicaria* (8), *Gossypium darwinii* (7), *Gossypium hirsutum* (8), *Kalanchoe laxiflora* (6), *Lotus japonicus* (7), and *Triticum aestivum* (7). At the same time, these species mainly exhibit the duplication of the MBF1a and MBF1b subfamilies. These results could explain one of the reasons why herbaceous plants are often much more resistant to abiotic stress.

### Gene structure and subcellular location analysis

**Fig. 2:**
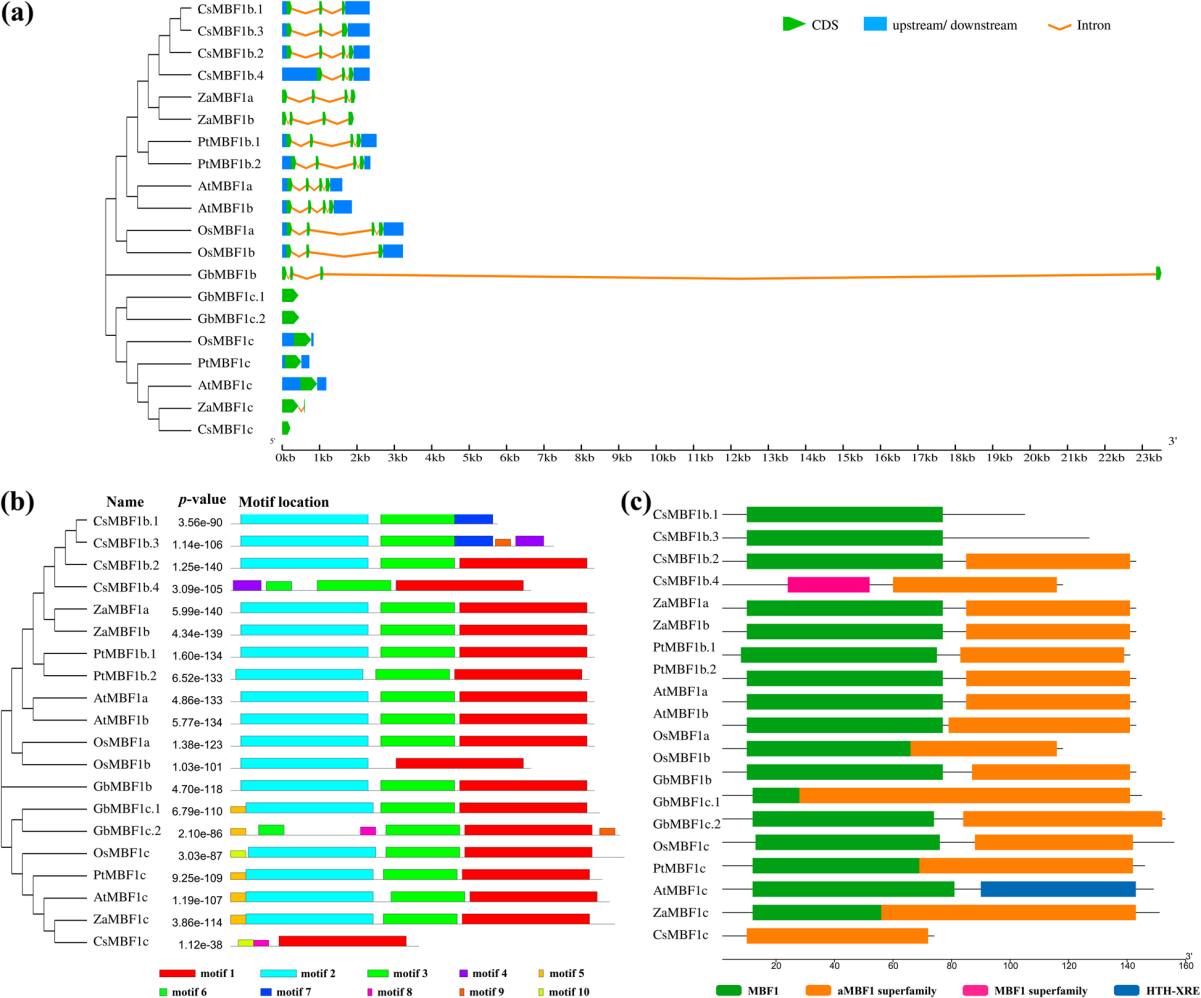
The phylogenetic and gene structure analyses of *MBF1* genes from six representative species. **a** The genomic structure of various *MBF1* genes. The blue box was UTR regions, green box was exons, and organe line was introns. The phylogenetic tree was also conducted using the amino acid sequences of MBF1 proteins in each species. **b** The motif distribution of each MBF1 member. **c** The conserved protein domain distribution of each MBF1 protein, aMBF1 was archaeal ribosome-binding protein aMBF1 domain

To comprehensively explore the information of the MBF1 family, the six species in this study were selected for gene structure analysis (Fig. 2). Similarly, the *MBF1* genes were clustered in two major branches. The MBF1a and MBF1b subfamilies belonged to the same class, and all of them had 2–3 introns and 3–4 exons (Fig. 2a). The exon lengths of MBF1a and MBF1b were similar in each plant, but longer introns were detected in *O. sativa* and *G. biloba*, especially *GbMBF1b*. Both *ZaMBF1a* and *ZaMBF1b* were showed four exons and three introns. Furthermore, most of the *MBF1c* genes only had one exon, but the CDS of *ZaMBF1c* was separated by a short intron. Additionally, the species with larger genomes (*G. biloba* and *Z. armatum*) do not exhibit UTR regions in their MBF1 members.

To further analyse the biological functions, the motif and conserved domains were identified in the twenty MBF1 proteins of various plants. Ten conserved motifs were identified in the MBF1 protein sequence by MEME software (Fig. 2b), and three significant motifs were detected and displayed in Additional file 4: Table S4. Motif 2 was discovered to be related to the MBF1 domain, which is involved in the aMBF1 superfamily (Fig. 2c, Additional file 5: Table S5). Motif 5 was only detected in the MBF1c subfamily in some species. Similar motifs and domains were detected in ZaMBF1a and ZaMBF1b, which indicated that they might endow some similar biological functions.

**Fig. 3:**
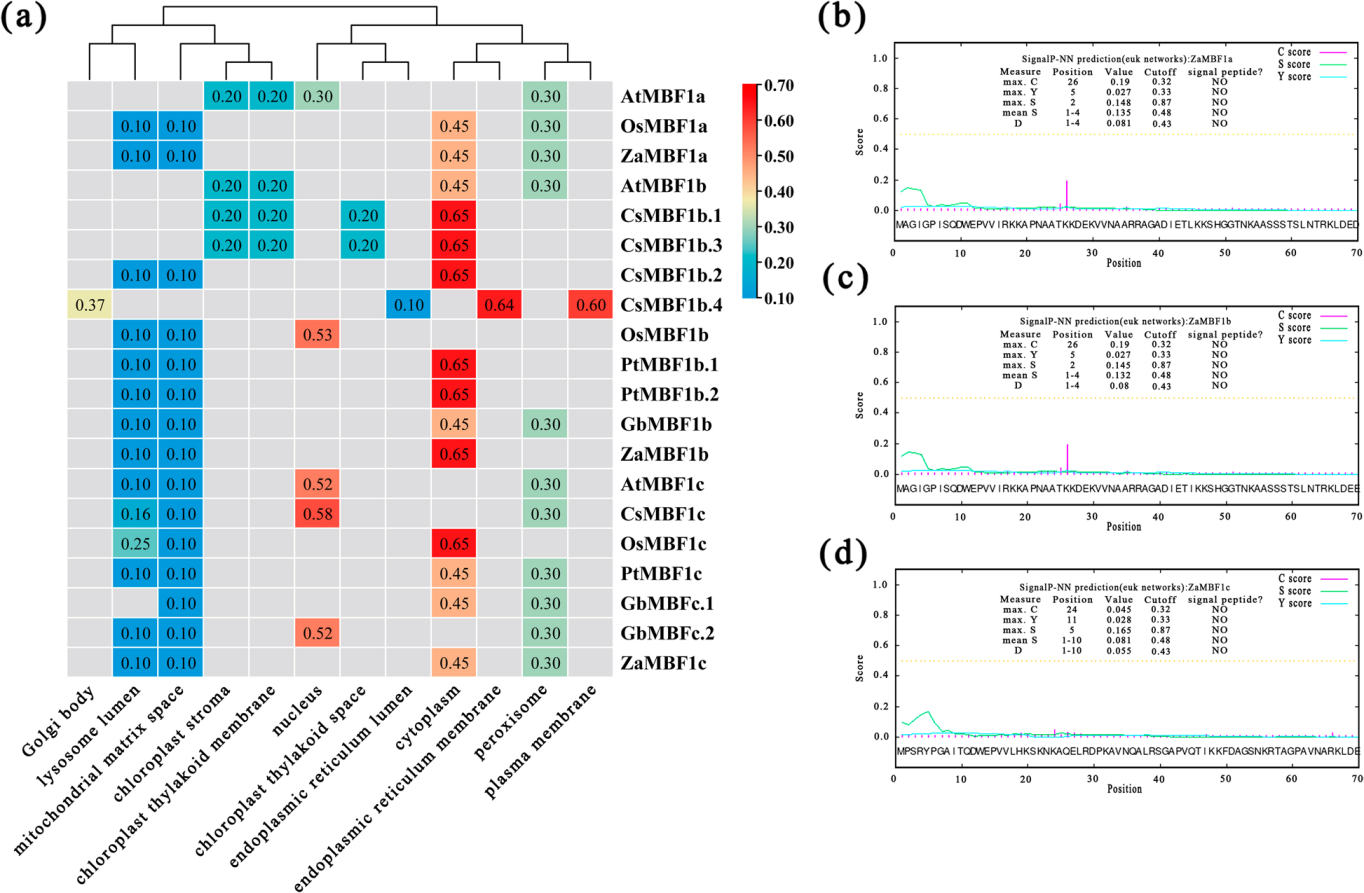
The subcellular localization and signal peptide prediction of MBF1 genes. **a** the subcellular localization of *MBF1* genes from six representative species, the data is the certainty of each MBF1 located in the various sites. **b**-**d** the signal peptide prediction of ZaMBF1 family

Moreover, subcellular localization predictions were made for all MBF1 genes of the six species (Fig. 3a). The results showed that most of them were located in the cytoplasm, and the rest were located in the nucleus, except CsMBF1b.4. All the ZaMBF1 were mainly located in the cytoplasm, especially ZaMBF1b. ZaMBF1a and ZaMBF1c could also be related to peroxisome, which might imply an important role as resistance factors. Moreover, the signal peptide prediction is additional evidence for the localization of genes. The results showed that no signal peptide sequence was detected in any of the three ZaMBF1 genes (Fig. 3b-d), which indicated that they were not secreted proteins or transmembrane proteins, so they could only be freed in the cytoplasm or localized in the nucleus. The consistent result in a previous study was that the subcellular localization of MBF1 was dependent on plant development stage, interaction with other transcription factors, and environmental conditions, and it could shuttle back and forth between the cytoplasm and the nucleus [14].

### Analysis of chromosomal locations and collinearity

The chromosomal location analysis showed that the MBF1 genes were closer together on chromosomes when their relationships were closer (Additional file 6: Figure S1). For example, the four *CsMBF1b* were located in nearby sites on CsChr5. *OsMBF1a* and *OsMBF1b*, *GbMBF1c.1* and *GbMBF1c.2* also displayed the similar located patterns. A very important finding was that three *ZaMBF1* genes were located on three different chromosomes. Although *ZaMBF1a* and *ZaMBF1b* showed the close gene structure and phylogenetic relationships, they were still observed on ZaChr26 and ZaChr32, respectively. This result again suggested that a gene duplication event might have occurred in *ZaMBF1a* and *ZaMBF1b*.

**Fig. 4:**
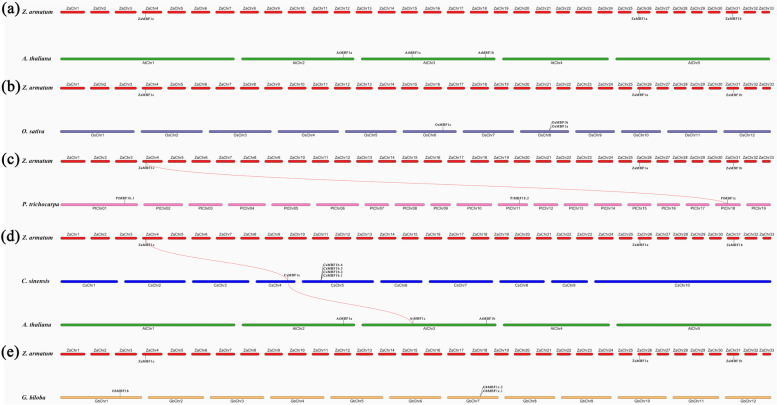
The synteny analysis of the *MBF1* genes between *Z. armatum* and other five species. The red lines highlighted the syntenic *MBF1* pairs between *Z. armatum* and the species

To confirm our results, we carried out collinearity analysis by the MCScanX method using the genome data of *Z. armatum*. The results showed that obvious collinearity occurred in the genomic profile of *Z. armatum*, and the three *ZaMBF1* genes also showed the gene replication events among each other (Fig. 4). The higher sequence similarity between repetitive gene pairs was due to the ZaMBF1 family involvement in the similar biological regulation processes, and the gene replications were likely to promote the evolution of the *ZaMBF1* genes in *Z. armatum*.

**Fig. 5:**
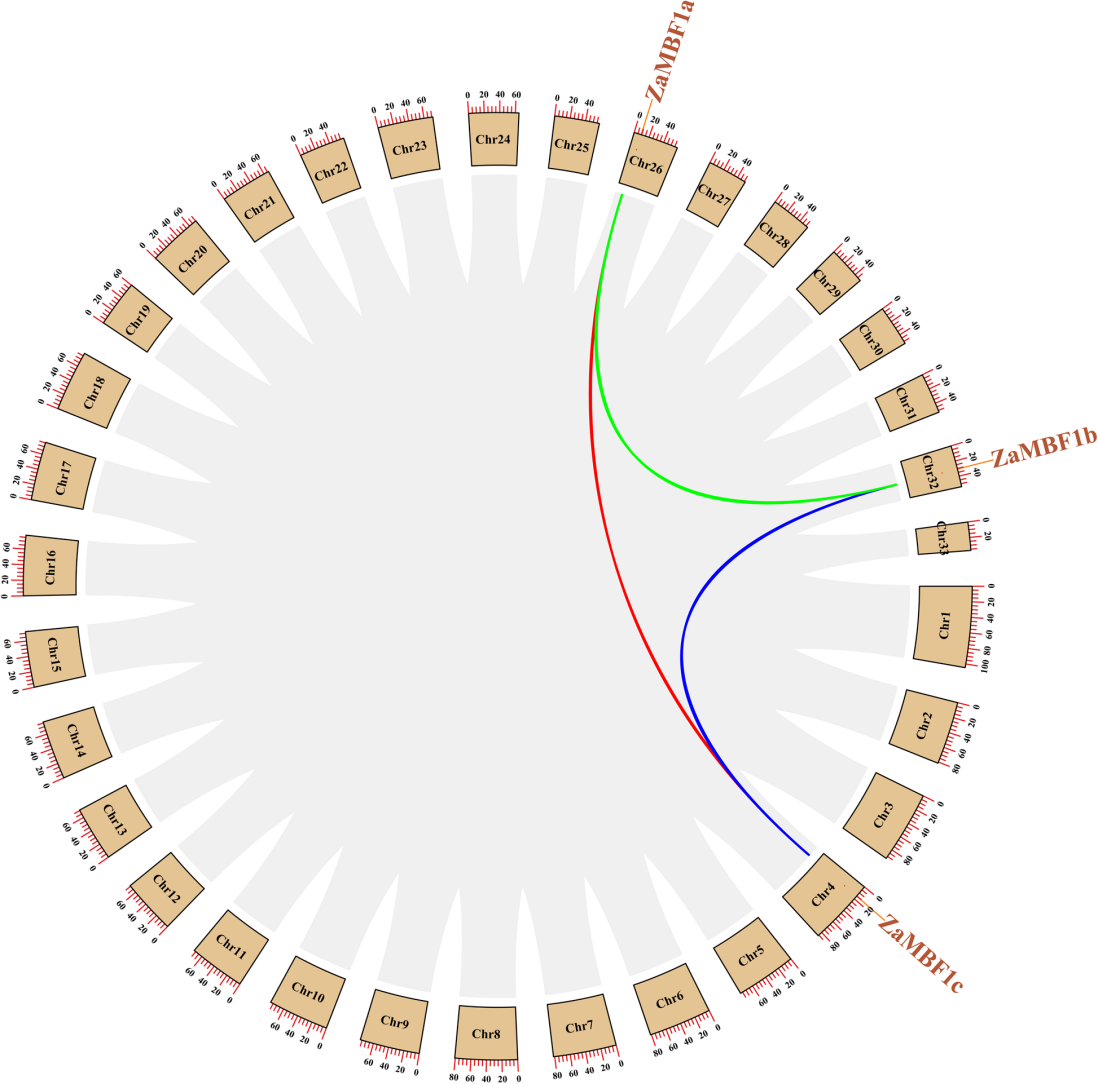
The schematic diagram of *ZaMBF1* distributions and syntenic relationships in *Z. armatum*. The gray lines were the entire syntenic blocks in chromosomes of *Z. armatum*. Red, green and blue lines present the duplicated *ZaMBF1* gene pairs in *Z. armatum*. The chromosome number was shown in the middle of the arc square lattice. The length of each chromosome was marked by the red scale (Mb)

To further analyse the evolutionary relationship of *MBF1* genes in the plants, the comparative syntenic maps were first constructed between *Z. armatum* and other species. The results showed that a rich synteny was detected between *Z. armatum* and either*A. thaliana* or *O. sativa*, but it was not present in *MBF1* family genes (Fig. 5a-b). However, the paralogous *MBF1* genes were investigated in the woody plants in this study (Fig. 5c-d). *MBF1c* acted as one pair of syntenic genes observed in *Z. armatum* and *P. trichocarpa* or *C. sinensis.* Surprisingly, *C. sinensis*, as a proximal relationship species, was also found to have a syntenic gene with *A. thaliana* (Fig. 5d). These results suggested that *MBF1c* may play a vital role in the evolutionary process of the MBF1 gene family, and some considerable differentiation of *MBF1* genes might have been carried out during the evolution of perennial woody plants. Meanwhile, few syntenic relationships were detected between *Z. armatum* and *G. biloba* (Fig. 5e), which might indicate significant differences between angiosperms and gymnosperms during the long history of evolution.

### Cis-elements and tertiary structure analysis of *MBF1*

**Fig. 6:**
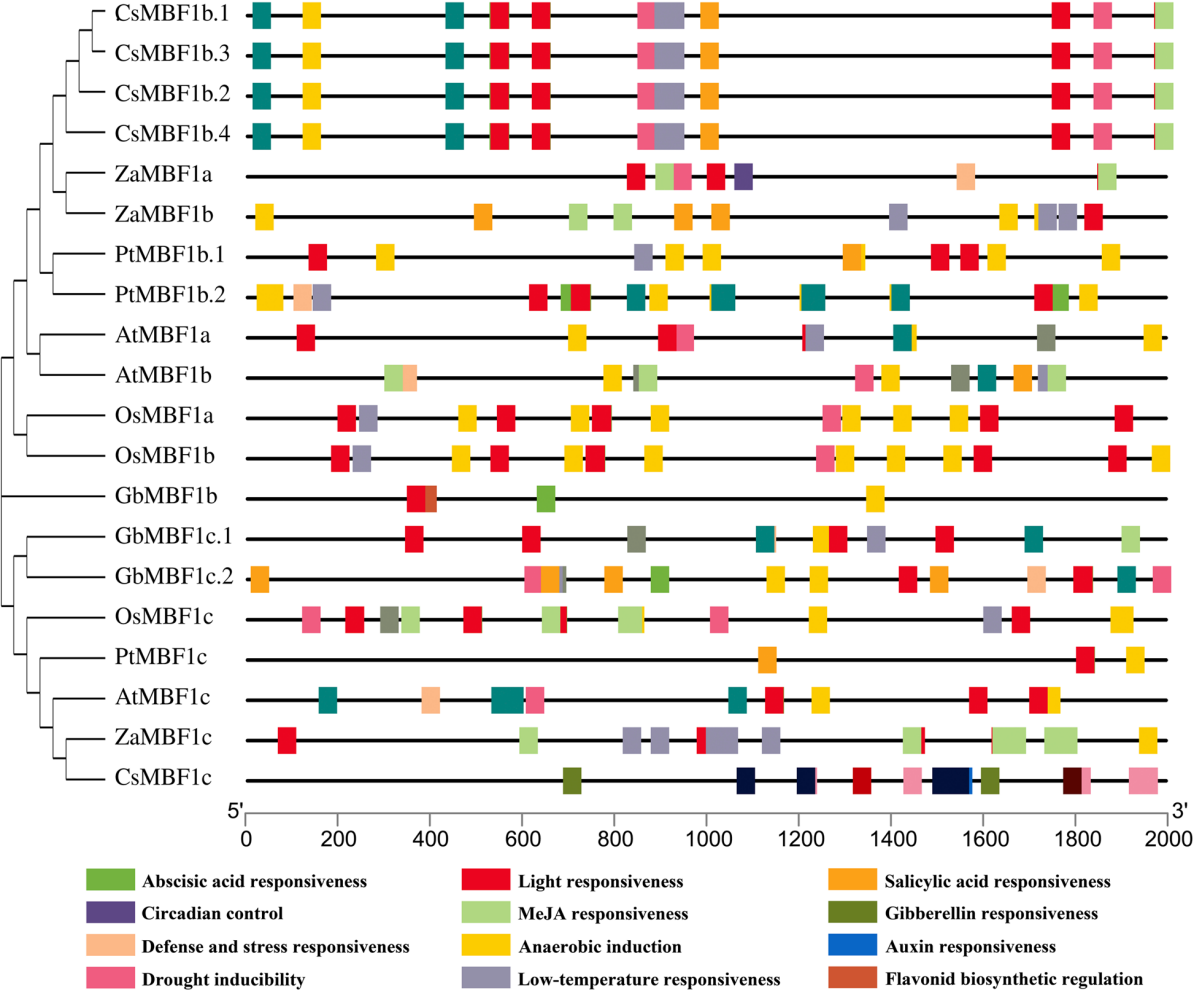
The cis-regulatory elements analysis of *ZaMBF1* promoter regions. The relative locations of stress, phytohormone and growth cis-elements in *ZaMBF1* promoter regions. The different colors were the various cis-acting elements, and their positions were marked in the corresponding promoter sequences

To further determine the molecular functions and expression pattern of the *MBF1* family, the promoter regions of 2000 bp upstream from the transcription starting site were isolated from the genomic data of six species. A series of cis-acting elements were identified as being associated with abiotic stress (Additional file 7: Table S6). Furthermore, most promoters exhibited the responses to drought, light, anaerobic, and low-temperature conditions (Fig. 6, Additional file 8: Table S7). The eight cis-elements detected in *ZaMBF1a* were mainly related to drought and light responsiveness. An exclusive cis-element associated with circadian control was obtained in *ZaMBF1a* among the *MBF1* promoters of six species. Meanwhile, 12 cis-elements were investigated from *ZaMBF1b*, which were mainly involved in responses to light, anaerobic and low-temperature induction. The cis-elements of *ZaMBF1c* were likewise focused on light, anaerobic induction and low-temperature responsiveness.

In addition, some phytohormone pathways related to stress were also detected in these promoter regions (Fig. 6, Additional file 8: Table S7). All of the species were detected to have at least one cis-element involved in abscisic acid (ABA) responsiveness in the present study, except *Z. armatum*. We also could not observe any cis-elements related to gibberellin (GA) responsiveness in *Z. armatum* and *O. sativa*. However, there were more cis-elements associated with MeJA and salicylic acid (SA) responsiveness in *Z. armatum*. These results indicated that the pathways of MeJA and salicylic acid might play vital roles in biotic and abiotic stress resistance in *Z. armatum*. Notably, almost every cis-element was detected in *G. biloba*, which might explain its strong vitality in the Mesozoic era.

**Fig. 7:**
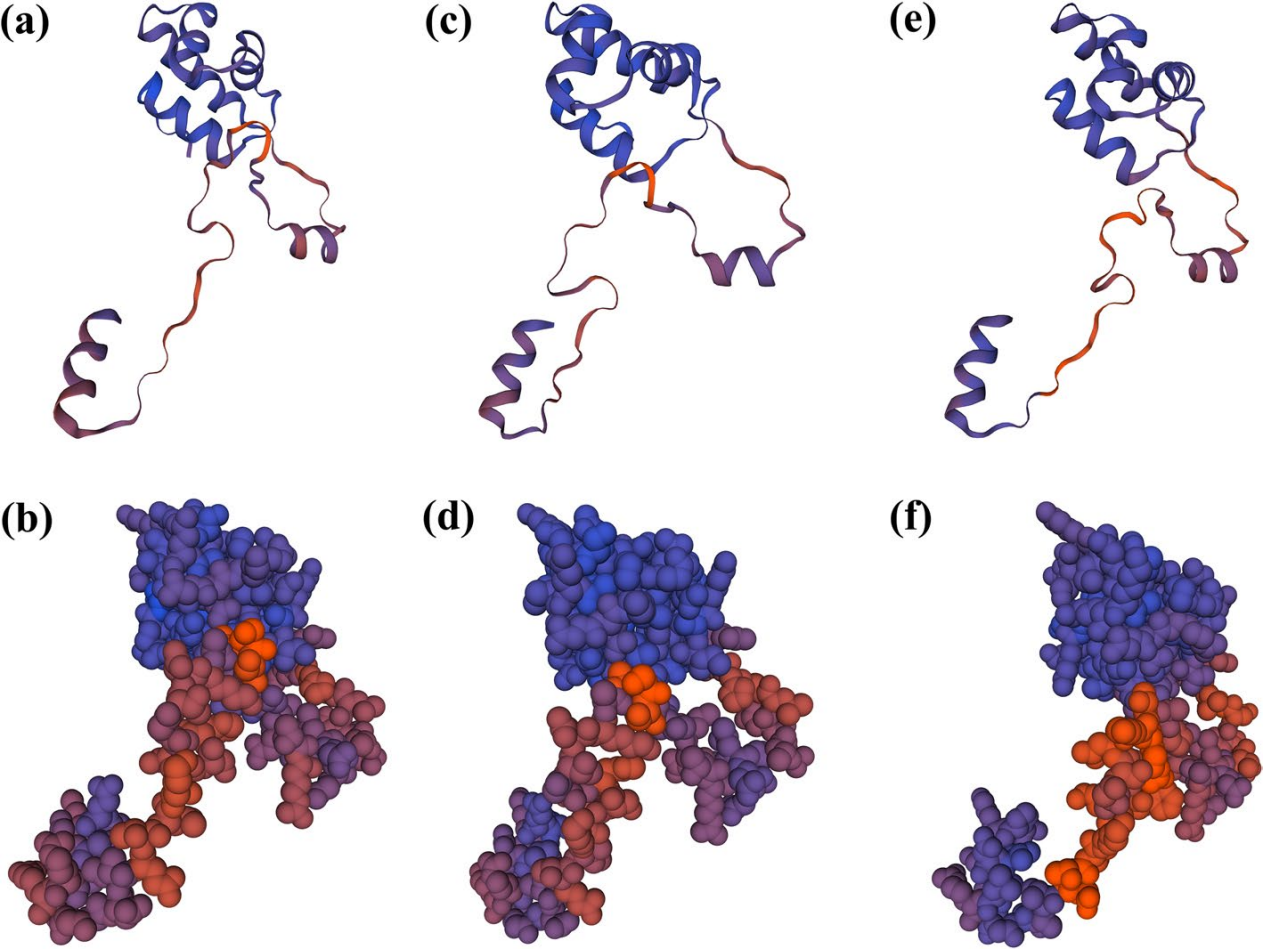
The predicted three-dimensional domains of ZaMBF1 proteins in *Z. armatum*. **a** The superposed structures of the ZaMBF1s in 3D, including *α*-helix, *β*-pleated sheet, *β*-turn and random coil. **b** The atom models of ZaMBF1s in *Z. armatum*

Moreover, the tertiary structures of the ZaMBF1 amino acid sequence were investigated by the SWISS-MODEL server (Fig. 7). Both the MBF1 domain and helix-turn-helix domain were detected in each protein. A similar 3D structure was observed in *ZaMBF1a* and *ZaMBF1b* (Additional file 9: Table S8), which suggested that these genes may perform shared functions. However, *ZaMBF1c* displayed a relatively different tertiary structure, especially in the helix-turn-helix domain.

### TF binding site analysis of the *ZaMBF1* family

**Fig. 8:**
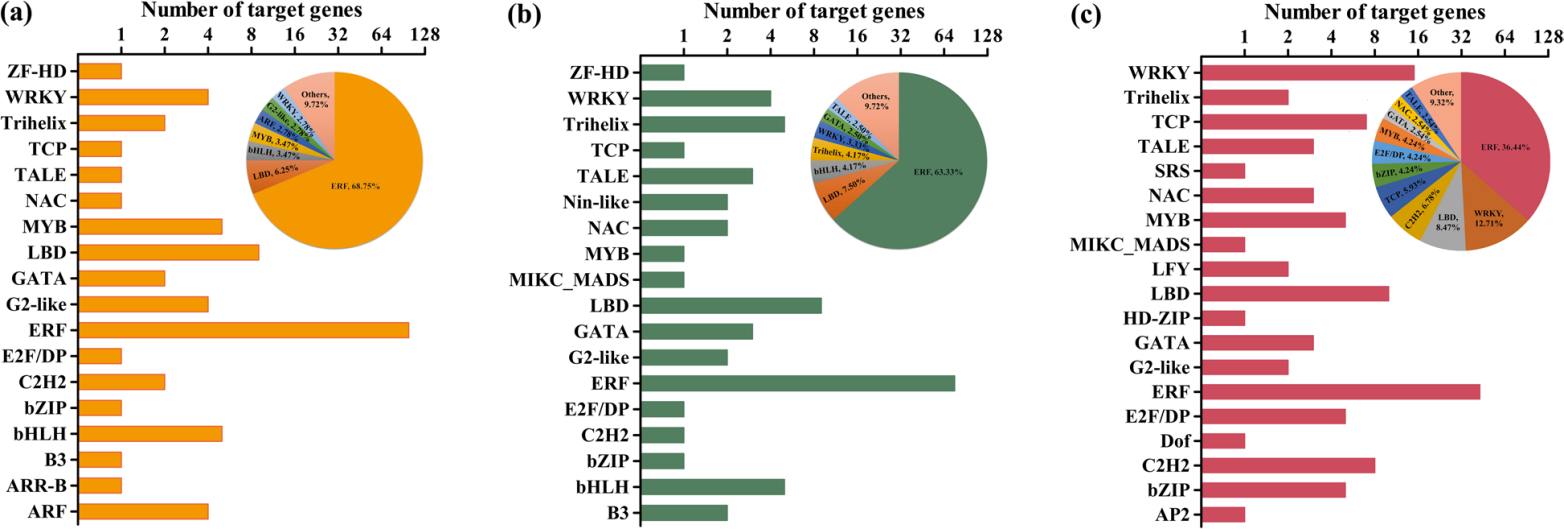
The transcription factor binding sites of ZaMBF1 members in *Z. armatum*. **a** The predicted information of the target genes interacted with ZaMBF1a. **b** The predicted information of the target genes interacted with ZaMBF1b. **c** The predicted information of the target genes interacted with ZaMBF1c. The pie charts were the percentage of the various transcription factors in each ZaMBF1 member, respectively

According to the results from the Plant Transcriptional Regulatory Map, a total of 382 TF binding interactions were observed in the *ZaMBF1* family, and their length ranged from 8 to 28 bp (Additional file 10: Table S9). Most of the target genes were associated with the responses to plant stress, such as the WRKY, Trihelix, NAC, MYB, LBD, and C2H2 (Fig. 8). The ERFs were the most frequent target sites in the sequences of the *ZaMBF1* family, which indicated that a strong interaction might be carried out between ERFs and ZaMBF1 proteins. Meanwhile, some ARR sites were also detected in *ZaMBF1a*, which are important members of the cytokinin signal transduction pathway. It is worth noting that some TF sites involved in floral differentiation were identified in *ZaMBF1c* (Fig. 8c), including one site related to MIKC_MADS, two sites related to LFY, one site related to Dof, and one site related to AP2. Meanwhile, a site related to MIKC_MADS was also detected in *ZaMBF1b.* These results suggested that the *ZaMBF1* family not only plays the vital roles in the response to stress, but also performs some important functions in the reproductive growth process.

### Expression patterns of the *ZaMBF1* family according to RNA-seq

**Fig. 9:**
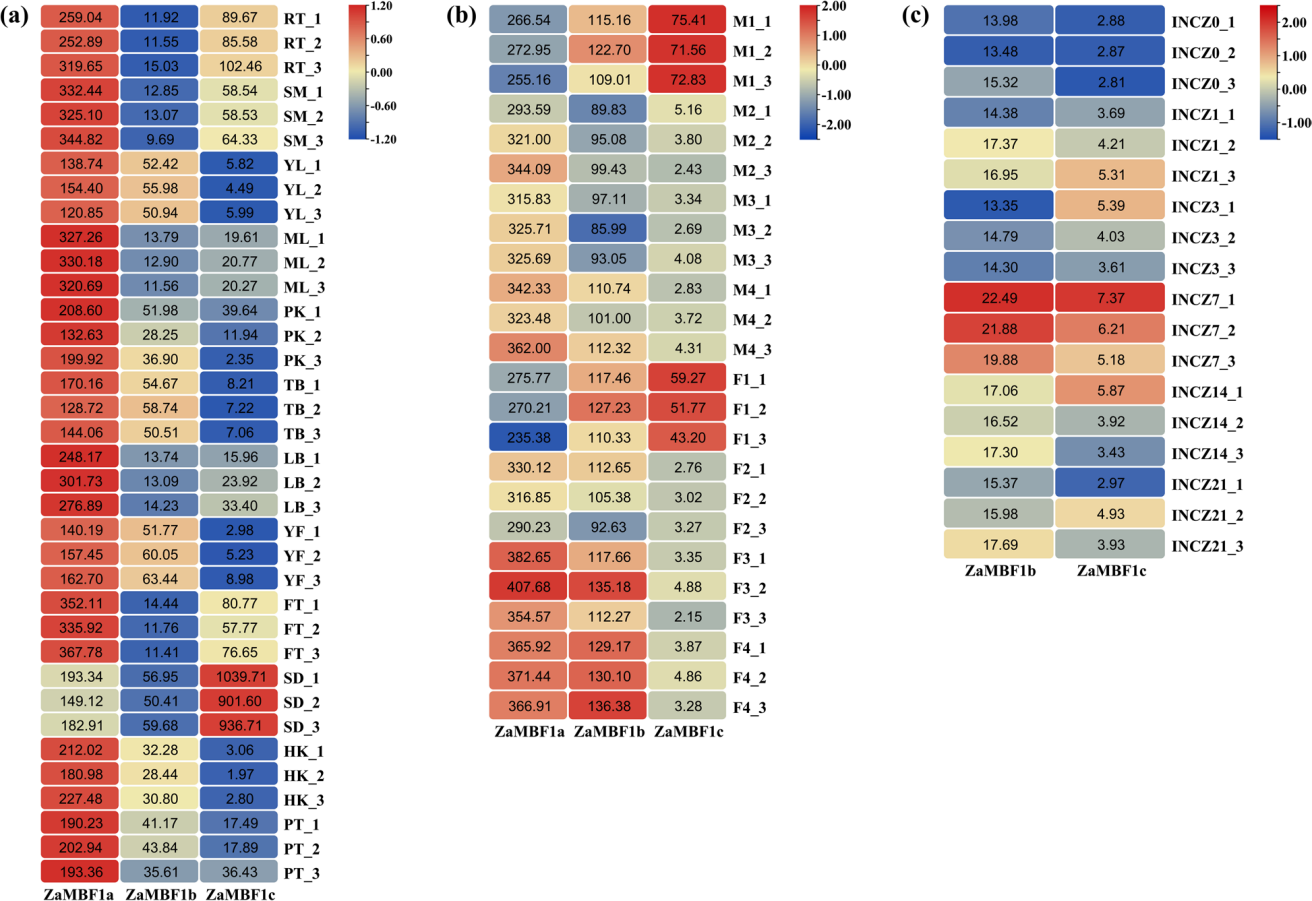
The expression profiles of *ZaMBF1* genes in different organs and biotic stress based on transcriptome dataset. **a** The expression heat maps of three *ZaMBF1*s in 12 different tissues involved in the vegetative organs and seeds. RT, Root; SM, Stem; YL, young leaf; ML, Mature leaf; PK, prickle; TB, terminal bud; LB, leaf bud; YF, young flower; FT, Fruit; SD, seeds; HK, husks; PT, petiole. **b** The expression abundance of *ZaMBF1* genes in floral differentiation and development process. The M1-M4 were the different samples in male floral differentiation process, and the F1-F4 were the different samples in female floral differentiation process. **c** The expression abundance of *ZaMBF1* genes after leaf rust disease infection. INCZ0, 0 days after infection; INCZ1, 1 days after infection; INCZ3, 3 days after infection; INCZ7, 7 days after infection; INCZ14, 14 days after infection; INCZ21, 21 days after infection. The color bars were the relative expression levels after column scale, and the data in each heatmap was the original FPKM values of the samples

To explore the role of the MBF1 family in the growth and development of *Z. armatum*, the expression abundance of MBF1 genes was investigated in the transcriptome dataset. Based on the expression profiles from the 12 various organs in *Z. armatum* (Fig. 9a, Additional file 11: Table S10), *ZaMBF1a* exhibited the opposite expression patterns as *ZaMBF1c*. *ZaMBF1a* was highly expressed in all of the samples except for seeds, and high abundances were observed in roots, stems, and mature leaves. This result suggested that *ZaMBF1a* plays a crucial role in the growth of *Z. armatum*. However, *ZaMBF1c* had a relatively high expression in seeds but a relatively lower expression in fruits, husks and other samples. This result indicated that *ZaMBF1c* might be associated with the seed development process. *ZaMBF1b* often showed moderate expression compared with *ZaMBF1a* and *ZaMBF1c*, which implied that its role in the growth and development of various organs might be redundant.

Meanwhile, similar expression patterns were still observed during the male and female floral differentiation processes (Fig. 9b, Additional file 12: Table S11). *ZaMBF1c* was significantly up-regulated in the initiation stage of floral sex differentiation, but down-regulated in the subsequent floral differentiation and development process. This result also confirmed that the *ZaMBF1c* conducts some important functions in the reproductive growth process. However, *ZaMBF1a* exhibited a complementary expression pattern in the male and female flower samples, but *ZaMBF1c* showed an opposite expression patterns. These results suggested that both *ZaMBF1a* and *ZaMBF1c* play critical roles in vegetative and reproductive development. Additionally, *ZaMBF1b* was not detected an obvious expression pattern, which indicated that it might be a redundant gene that serves as a supplementary functional member in the growth and development of *Z. armatum*.

To further understand the role of the *ZaMBF1* family in response to stress, we performed the expression patterns of *ZaMBF1* genes after infecting the leaves with rust disease (Fig. 9c). Both *ZaMBF1b* and *ZaMBF1c* were observed to be significantly up-regulated in the 7 days after infection (INCZ7), which suggested that this time was a vital point for *ZaMBF1* genes to respond to leaf rust disease. However, we could not find any information about the *ZaMBF1a* gene in the transcriptome dataset after infection. This result indicated that the expression of *ZaMBF1a* may not be significantly induced by leaf rust disease. All of these results will be helpful for future research on gene function and molecular mechanisms.

### Expression profiles of the *ZaMBF1* family in response to different treatments

**Fig. 10:**
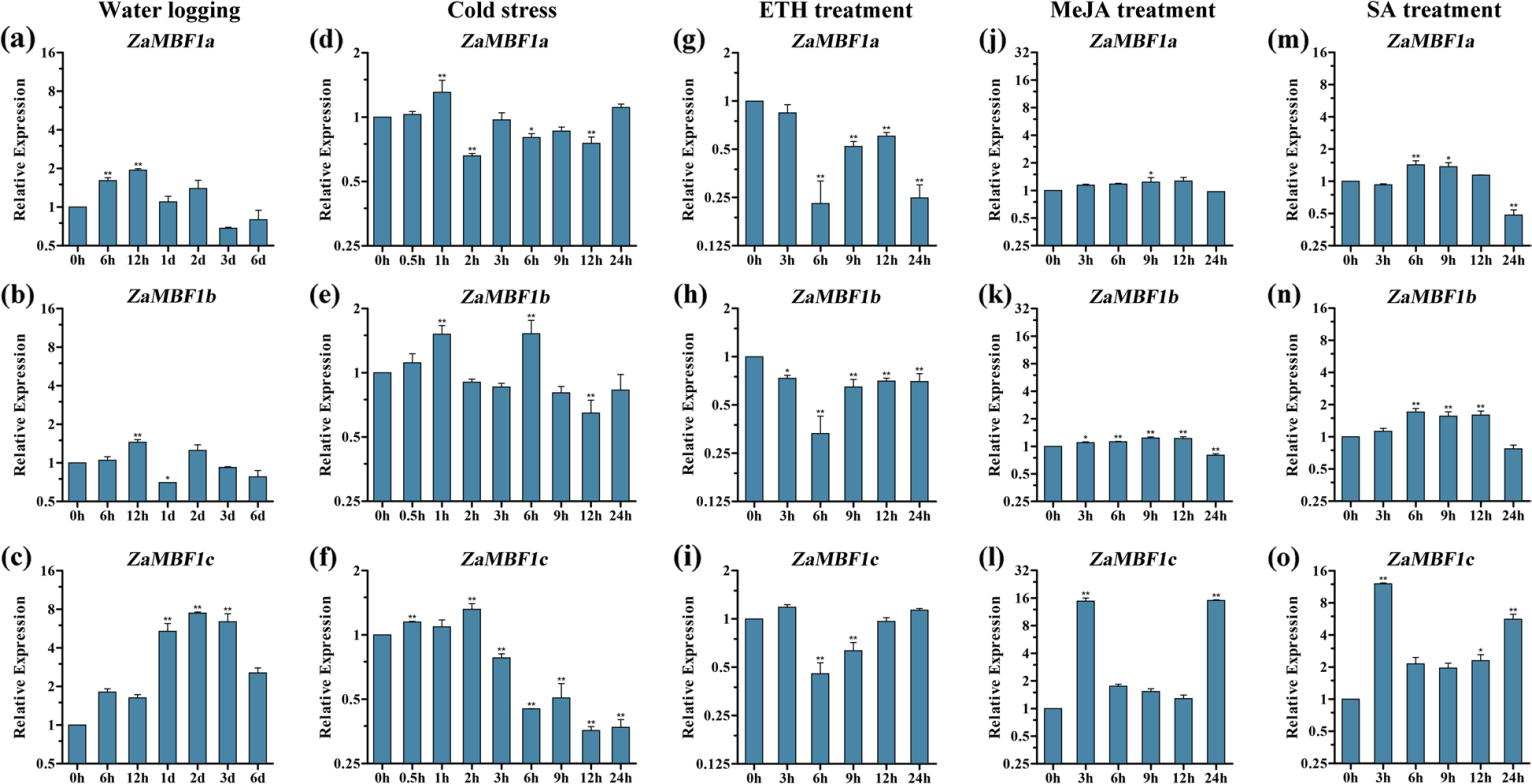
The qRT-PCR analysis of the expression patterns associated with *ZaMBF1* genes under different stress treatments. (**a-c**) water logging treatment, (**d-f**) cold treatment, (**g-i**) ethephon treatment. (**j-l**) MeJA treatment, (**m–o**) SA treatment. The 0 h was selected as the control sample to estimate the relative expression in each treatment, and the asterisks were the significant differences, * indicated the *p* < 0.05; ** indicated the* p* < 0.01

To deeply uncover the roles of the *ZaMBF1* family in response to stress, various treatments were carried out to detect the expression of three *ZaMBF1* genes by qRT-PCR (Fig. 10). Totally, each treatment could significantly induce or inhibit the expression of *ZaMBF1* genes in *Z. armatum*.

Similar expression patterns were observed in *ZaMBF1a* and *ZaMBF1b* after water-logging treatment (Fig. 10a-c). Both of them were up-regulated at 6 and 12 h, but gradually down-regulated to the normal level (similar to 0 h) in the subsequent days. However, *ZaMBF1c* was found to have a very significant up-regulation after water-logging treatment (Fig. 10c), the expression of which was approximately eightfold higher after two days treatment compared to the baseline at 0 h. Even though the seedlings were treated 3 days with water-logging, a very high relative expression of *ZaMBF1c* was still detected. These results suggested that the *ZaMBF1* family could play an important role in the response to water-logging stress, especially the *ZaMBF1c* gene.

Additionally, there was no significant pattern in the expression of *ZaMBF1a* and *ZaMBF1b* after cold treatment (Fig. 10d-f). However, *ZaMBF1c* was very significantly down-regulated in the cold environment, and was significantly inhibited after 6 h of treatment (Fig. 10f). These results implied that *ZaMBF1a* and *ZaMBF1b* might not be directly related to cold stress, while *ZaMBF1c* showed a negative response to cold stress in *Z. armatum*.

Due to the crucial target sites of ERFs in the sequences of the *ZaMBF1* family, ethephon (ETH) was selected to treat the seedlings and investigate the expression patterns of the three *ZaMBF1* genes (Fig. 10g-i). The confirmatory results showed that significant down-regulation was observed at 6 h after ETH treatment among the three *MBF1* genes, especially the *ZaMBF1a* genes, which had more ERF target sites (Fig. 8). However, the expression were gradually up-regulated to the normal level by 9 h after ETH treatment. All of these results suggested that negative regulation might also be used to respond to ETH signalling in the *ZaMBF1* family, and that *ZaMBF1a* might be a vital member due to its enriched ERF elements.

The effects of MeJA treatment showed that *ZaMBF1a* and *ZaMBF1b* had comparable similar expression patterns (Fig. 10 j-l), and they were gradually up-regulated within the 12 h posttreatment and returned to normal levels at 24 h posttreatment. However, *ZaMBF1c* was found to be very significantly up-regulated at 3 h posttreatment (Fig. 10 l), and very high expression was also detected again at 24 h after treatment. These results indicated that the *ZaMBF1* genes also play an important role in the response to MeJA stress, especially the *ZaMBF1c* gene.

To confirm the information of the *ZaMBF1* family response to phytohormones involved in plant stress resistance, SA treatments were also conducted to detect the expression of the three *ZaMBF1* genes (Fig. 10 m–o). *ZaMBF1a* was significantly induced within the 9 h of treatment, but significantly down-regulated at 24 h after treatment. Similar results were observed for *ZaMBF1b.* However, significant up-regulation was observed in the expression of *ZaMBF1c* after SA treatment (Fig. 10 o), and the expression level of 3 h after treatment was approximately 12-folds higher than that of the control. To sum up, the three *ZaMBF1* genes could serve their functions in response to plant stress via phytohormone signalling pathways. Both *ZaMBF1a* and *ZaMBF1b* performed their functions within 12 h after treatment, whereas the *ZaMBF1c* could continue to work via their powerful functions in the plant stress resistance.

## Discussion

Multiprotein Bridging Factor 1 (MBF1) is an ancient super-family across plant life, that has a typical helix-turn-helix structure [14]. The first study of MBF1 was reported from silkworm as a non-DNA binding transcription co-factor [35]. In the present study, we selected MBF1 from six representative species to uncover its characteristics. Compared with *A. thaliana*, all other species have at least two different *MBF1* genes, and *MBF1c* is an essential member. Interestingly, the CDS length of most *MBF1c* was slightly longer than that of the *MBF1a* and *MBF1b* genes in each species. MBF1a and MBF1b were more closely relationship, whereas MBF1c displayed relatively independence according to the phylogenetic analysis of 202 amino acid sequences from 53 species. The consistent results were that MBF1 family could be divided into two groups, and the structure of Group I was four exons and three introns. The structure of group II contained zero, one, or two introns [14]. Thus, MBF1a and MBF1b could be deemed to belong to the Group I subfamily, and MBF1c could be deemed to belong to the group II subfamily in the current study. This might be the reason why the MBF1a and MBF1b subfamilies were alternately clustered into a large group in the present study (Fig. 1a). In addition, the gene structure with few or no introns might have contributed to rapid transcription and translation to enhance the ability to respond to external stress [36, 37]. This might explain why *G. biloba*, a famous living plant fossil, survived the ice age due to an increased number of MBF1c members. Furthermore, the short sequence of *MBF1c* might be caused by the limited genome assembly in *C. sinensis*. It is worth noting that the gene structures of *ZaMBF1a* and *ZaMBF1b* appeared to be symmetrical and obtained more similar motifs and domains (Fig. 2), and collinearity was also observed between *ZaMBF1a* and *ZaMBF1b* (Fig. 4), all of which suggested that a gene duplication event occurred in *ZaMBF1a* and *ZaMBF1b*. Gene replication events, which usually depended on tandem and segmented replications, could promote the expansion of gene family members and enhance the potential regulation abilities to adapt to the various environmental changes [38–40]. Previous studies suggested that tandem repeats might provide some similar genetic functions and expression patterns [37, 41]. Thus, the detailed functions and the essential members of these *ZaMBF1* genes could be revealed in further studies.

The expansion of gene family members and the mechanism of genomic evolution were mainly due to gene duplication events, including tandem and segmented replications [37]. Duplication events related to the resistance gene families have been reported in many plant species [1, 37, 41], but there have been no findings related to the MBF1 family. In this study, three *ZaMBF1* genes were located on different chromosomes without any tandem duplication, whereas the obvious collinearity was present among them. In a previous study, an independent whole genome duplication (WGD) event was followed by extreme amplification of transposable elements in *Z. armatum*, which promoted the genome size expansion of *Z. armatum* [34]. Thus, gene replication or WGD events might have contributed to the evolution of *ZaMBF1* genes in *Z. armatum* [42, 43]. Additionally, the analysis of collinear regions between species suggested that *ZaMBF1c* exhibited collinearity only with the *MBF1c* genes in *P. trichocarpa* and *C. sinensis*. These results indicated that the orthologous genes may distinctly evolve in similar species types, which was consistent with previous studies on the collinearity of WRKY genes in pine/apple/rice/corn and NAC genes in *A. thaliana*/apple/grape/rice [1, 44]. Meanwhile, Zanthoxylum is relatively recent genus and diverged from Citrus approximately 36.5–37.7 million years ago [45]. *Z. armatum* and *C. sinensis*, both of them belonging to the Rutaceae family, were estimated to have diverged approximately 32.9 million years ago, and *Z. armatum* experienced an independent whole-genome duplication event approximately 26.6 MYA after its split with Citrus [34]. Thus, these reasons might result in the syntenic gene pairs of *MBF1c* obtained in *Z. armatum vs. C. sinensis* and *C. sinensis vs. A. thaliana*, but not between *Z. armatum* and *A. thaliana*. Furthermore, the syntenic region of *MBF1c* was also present between *A. thaliana* and *C. sinensis*, but was not detected among *ZaMBF1c* with *O. sativa* and *G. biloba*, implying that *MBF1c* developed after the divergence of dicots and monocots under the clade of angiosperms [1].

The transcription factor binds to cis-elements located in the gene promoter sequences to play their core roles regulating the expression of the genes and controlling the growth, development, and responses to the environment in plants [46, 47]. The structure of the promoter could suggest the possible functions and regulatory mechanism of the genes [3]. Furthermore, the copy number of the cis-elements could also determine the transcription level and cause the specific phenotype [48]. Our present study investigated cis-elements associated with *MBF1* in six different species. Fortunately, a total of 255 cis-elements were detected in six representative species, including a response to drought inducibility, light, anaerobic conditions, and so on. The highest number of cis-elements was observed in *O. sativa* and *A. thaliana*, implying that Poaceae and herbage have multiple stress resistance pathways. Additionally, most species have cis-elements involved in phytohormone pathways, such as the responsiveness to ABA, GA, MeJA, and SA, which suggested that MBF1 could interact with plant hormones to defend against plant stress [15, 19, 20]. Notably, almost every cis-element can be detected in *G. biloba*, which might explain why this species could have survived the long evolution of the Mesozoic era to become a living plant fossil. Additionally, a previous study found that *MBF1s* could regulate the expression of *ABA REPRESSOR1* (ABR1) under MV-induced (Methylviologen) stress [15, 19, 49]. The ectopic expression of *HbMBF1a* enhanced ABA insensitivity in *A. thaliana* [50]. However, we could not detect any cis-elements related to ABA and GA responsiveness in the promoter sequences of *Z. armatum*, suggesting that the *ZaMBF1* genes are not involved in the plant stress response process via ABA and GA pathways. Thus, detailed information on the *ZaMBF1* genes needs to be explored in further studies by exogenous induction, expression detection, and functional identification in *Z. armatum*.

Due to the necessity of flooding and cold resistance during the cultivation of *Z. armatum* and the detection of cis-elements associated with anaerobic induction and low-temperature responsiveness in *ZaMBF1* promoter regions, these abiotic stresses were selected to treat the seedlings and carry out the expression pattern analysis of *ZaMBF1* genes. Meanwhile, more MeJA and SA cis-elements were detected in the promoter regions and the crucial target sites of ERFs in the sequences of the *ZaMBF1* family in *Z. armatum*, so these factors were also chosen to investigate the changes in abundance of the *ZaMBF1* family in the present study. In a previous study, the ethylene response factor repressed *MBF1* expression by binding to its promoter region [51]. The consistent result was that the expression of *ZaMBF1* genes was very significantly down-regulated at 6 h but gradually up-regulated to the normal level in the subsequent times after ETH treatment. Thus, negative regulation might occur to respond to ETH signal in *ZaMBF1* family, especially for ZaMBF1a, due to the high frequency of ERF binding sites in its sequence. Similar to previous studies, the 6 h point after various treatments was a crucial point in the *ZaMBF1* genes response to external induction in the current investigation [20]. *ZaMBF1c* was observed to be more sensitive to these treatment and exhibited significant resistance, even after 3 days. In fact, *MBF1c* is a vital member that responds to the environmental changes in various species [19, 22, 49] and can interact with *TPS* gene family to defend against environmental stress [52]. Moreover, the function of *MBF1* genes has been of great interest in many plants [18, 19, 22, 53, 54], but they appeared to have different roles in the responsiveness to cold stress [14]. The ectopic expression of *AtMBF1* did not influence the response to cold stress in *Arabidopsis* seedlings [17], and a similar result was also observed in *R. raetam* [55]. The transcript abundance of *BocMBF1* could be significantly activated in *Brassica oleracea* [56]. However, a significant down-regulation was detected as the cold stress response in the overexpression of *CaMBF1* in hot pepper [15]. The consistent result was that *ZaMBF1b* and *ZaMBF1c* were very significantly down-regulated after cold stress in the current study, which is probably because both possessed more cis-elements associated with low-temperature responsiveness in their promoter regions. These results indicated that negative regulation may exist in response to low-temperature in *Z. armatum* [15]. Recently, water stress has seriously affected plant growth with global warming, which is one of the fatal factors in the worldwild decline in agricultural and forestry productivity [57]. Plants activate the antioxidant defence system and synthesize many stress response genes involved in signal transduction networks to avoid cell damage and improve their tolerance [58–60]. In previous studies, the overexpression of *MBF1* resulted in the accumulation of fewer reactive oxygen species and increased antioxidant enzyme activity [14]. The ability to contend against oxidative stress is severely impaired in *mbf1abc* triple mutant plants of *Arabidopsis*, and the roots and cotyledons of this triple mutant undergo cell death after treatment with H_2_O_2_ [61]. Fortunately, the consistent results in the present study were that all of the *ZaMBF1* genes showed the powerful responsiveness to the water logging treatment, especially the *ZaMBF1c* genes. However, the regulatory functions of *ZaMBF1* family are poorly understood. Thus, we suggest that these genes could be further researched to reveal their functions and molecular mechanisms related to anaerobic and cold tolerances, which would benefit the breeding of some high-quality varieties for perennial rainy areas in further studies.

Moreover, *MBF1* plays a significant role in growth development and stress tolerance and shows ubiquitous patterns of expression in various organs [14]. In previous studies, the MBF1 genes were detected in roots, stems, leaves, flowers, fruits, seeds and other tissues of various species [13, 15, 16]. However, the expression levels of three *MBF1* genes showed significant diversity in different tissues [14]. In the present study, the consistent results were that *ZaMBF1a* is predominantly expressed in all tissues except seeds, and seeds also showed a relatively high expression compared with that in other organs. This implied that *ZaMBF1a* might be an essential member associated with plant development. Meanwhile, *ZaMBF1c* was very significantly up-regulated in seeds but not in fruits and husks, suggesting that *ZaMBF1c* mainly participates in reproductive organ development. The confirmed results were that *ZaMBF1c* was significantly up-regulated in the initiation stage of floral sex differentiation, but extremely down-regulated in the subsequent floral differentiation and development process. Interestingly, we not only detected the target genes associated with the stress response in the sequence of *ZaMBF1c*, but also identified some target genes related to floral differentiation in the present study (Fig. 8). Thus, we suggest that *ZaMBF1c* could be triggered to improve plant stress tolerance and simultaneously induce the transition from vegetative growth to reproductive growth by activating *LFY*, *Dof*, and other MADS-box genes, when the plants suffer environmental stress. This regulatory mechanism could contribute to preserving their genotypes and rescuing the plants from disadvantageous conditions.

To sum up, our study carried out a genome-wide investigation of the *MBF1* family in six representative species. A series of bioinformatics analyses were performed in the present study, and the expression patterns of *ZaMBF1* genes in response to various external abiotic stresses and phytohormone inductions were investigated by qRT-PCR analysis. *ZaMBF1a* might be an essential member for plant development, and *ZaMBF1b* may serve redundant functions for *ZaMBF1a* in *Z*. *armatum*. Additionally, *ZaMBF1c* played an important role during the reproductive organ development process, and it also demonstrated strong responsiveness to environmental changes. However, our study identified only the *MBF1* genes to provide comprehensive bioinformatics results based on genome-wide and transcriptome profiles. Future studies should focus on generating transgenic *Zanthoxylum* lines to reveal the molecular functions of *ZaMBF1* genes [22]. The proteins that interact with ZaMBF1 could be explored by yeast two hybridization, bimolecular fluorescence complementarity and co-immunoprecipitation assay technique [52]. Promoter identification could also be conducted to uncover the genetic information of *ZaMBF1c* related to floral meristem differentiation in *Z*. *armatum*. Moreover, the content of various antioxidant enzymes and morphological characteristics could also be investigated in the transgenic *Zanthoxylum* lines [21], or even RNA-seq and metabolomics are an effective approach to isolate vital genes interacting with *ZaMBF1* [50]. All the above studies will help to reveal the molecular functions and regulatory mechanisms of *ZaMBF1* genes, which will contribute to breeding high-quality varieties of *Z*. *armatum* with meaningful stress tolerance.

## Conclusion

In this study, we identified *MBF1* genes in six representative species and characterized their gene structures, phylogenetic relationships, motif compositions, chromosomal distributions, *cis*-acting elements, gene duplications, and collinearity analysis. All the species have at least two different *MBF1* genes, and *MBF1c* is usually an essential member in most species. The gene replications occurred among three *ZaMBF1* genes, and *ZaMBF1c* showed collinearity between *Z. armatum* and both *P. trichocarpa* and *C. sinensis*. A total of 255 cis-elements involved in abiotic stress and phytohormone pathways were identified in the promoter regions of six species, which may reflect the response pathways of the *MBF1* family in each species. Moreover, the ERF binding sites were the most frequently targeted in the sequences of the *ZaMBF1* family, and *ZaMBF1c* may play a role promoting the transition from vegetative to reproductive growth in *Z*. *armatum*. Furthermore, *ZaMBF1a* is an essential member in various organs in *Z*. *armatum*, and *ZaMBF1c* might be associated with the male and female floral initiation processes. In addition, all of the *ZaMBF1* genes could be significantly induced by water-logging, cold stress, ethephon, methyl jasmonate, and salicylic acid treatments, especially *ZaMBF1c*. Our findings will boost functional genomics research on stress resistance genes in *Zanthoxylum*. Further studies could focus on confirming the genetic roles of these genes in breeding high-quality varieties with stress resistance in *Z*. *armatum*.

## Materials and methods

### Retrieval of *MBF1* genes in various species

The genome data of *Arabidopsis thaliana* (Athaliana_447_TAIR10) [62], *Oryza sativa* (Osativa_323_v7.0) [63] and *Populus trichocarpa* (Ptrichocarpa_533_v4.1) [64] were obtained from the Phytozome database (https://genome.jgi.doe.gov/portal/pages). The genome data of *Citrus sinensis* (C.sinensis_v2.0_HZAU) were obtained from the Citrus Genome Database (https://www.citrusgenomedb.org) [65]. The genome data of *Zanthoxylum armatum* were obtained from the FigShare database (https://doi.org/10.6084/m9.figshare.14400884.v1) [34]. The genome data of *Ginkgo biloba* were obtained from the Genome Warehouse database (https://ngdc.cncb.ac.cn/gwh/Assembly/18742/show) [66]. The transcription factors of each species were identified by iTAK_v1.6a software (https://github.com/kentnf/iTAK/releases) [67]. Furthermore, the *Multiprotein Bridging Factor 1* (*MBF1*) genes of each species were screened, and both the CDS and DNA sequences were isolated using Seqkit software according to their corresponding gff3 files (https://github.com/shenwei356/seqkit) [68]. Moreover, molecular weight (MW), isoelectric point (pI), and hydrophilicity analyses were performed by the ExPASy server and Protein GRAVY software (https://web.expasy.org/protparam). The *MBF1* genes of various species were annotated with the TAIR11 database (https://www.arabidopsis.org) and Pfam database (http://pfam.xfam.org/search#tabview=tab1).

### Evolutionary relationships of *MBF1* proteins

A total of 202 MBF1 proteins from 53 species were downloaded from the Phytozome database [69]. All the protein information of various species is shown in Additional file 1: Table S1, and they all have MBF1 and HTH conserved domains. The neighbour-joining phylogenetic analysis of all the MBF1 proteins was carried out using MEGA6.0 software with 1000 bootstrap replicates [70].

### Gene structure and subcellular localization analysis

To comprehensively analyse the information of the MBF1 family, six species, including *A. thaliana* (Herbage), *O. sativa* (Poaceae), *P. trichocarpa* (Xylophyta), *C. sinensis* (Homology), *G. biloba* (Gymnosperm) and *Z. armatum*, were selected for gene structure analysis. The introns, exons, and UTR regions of each MBF1 gene were investigated by the Gene Structure Display Server (http://gsds.gao-lab.org) [71]. The conserved motifs of each MBF1 protein were identified by MEME_v5.4.1 software (https://meme-suite.org/meme/tools/meme) [72]. The domains of each MBF1 protein were obtained from the CDD database (https://www.ncbi.nlm.nih.gov/Structure/bwrpsb/bwrpsb.cgi?) [73], and the conserved domains were drawn by TBtools software [74]. The subcellular localization of MBF1 genes were investigated by POSRT prediction software (http://psort1.hgc.jp/form.html), and the signal peptide prediction was carried out using the SignalIP-3.0 software (https://services.healthtech.dtu.dk/service.php?SignalP-3.0).

### Chromosomal locations, gene duplication and collinearity

In the present study, the chromosomal positions of each MBF1 gene in six species were collected from their genome data. The location of each MBF1 gene was displayed by TBtools software [74]. MCScanX software was applied to analyse *MBF1* gene duplication and collinearity events [75]. The threshold was set as follows: the E-value was 1e-10, and the alignment number was 5.

### Cis-regulatory elements and protein 3D structure analysis

The sequence of 2000 bp upstream from the transcription starting site of each *MBF1* gene was isolated by Seqkit software (https://github.com/shenwei356/seqkit), which was used to conduct cis-regulatory element analysis as promoter region. The cis-regulatory elements were investigated by the Plant-CARE database (http://bioinformatics.psb.ugent.be/webtools/plantcare/html/) [76]. All cis-regulatory elements associated with abiotic stress and phytohormones were identified and displayed by TBtools software [74]. Moreover, the tertiary structures of the ZaMBF1 protein were investigated by the SWISS-MODEL server (https://swissmodel.expasy.org/) [77].

### Transcription factor binding site analysis of the *ZaMBF1* family

To further clarify the transcription factor (TF) binding sites in the *ZaMBF1* family, the target genes were scanned by the Plant Transcriptional Regulatory Map website (http://plantregmap.gao-lab.org/) with the Binding site prediction tool. Due to the lack of a genomic dataset in this software, the homologous species, *C. sinensis*, was selected to perform this project.

### Transcriptomic data analysis of the *ZaMBF1* expression patterns

To explore the expression patterns of *ZaMBF1* in *Z. armatum*, the available transcriptomic data were collected from the NCBI database [34] and our studies [78]. A total of 26 different tissues were collected to analyse the *ZaMBF1* expression profiles in *Z. armatum*. The young flower (YF), young leaf (YL), husks (HK), seeds (SD), terminal bud (TB), petiole (PT), and prickles (PK) were obtained from public transcriptome data (BioProject: PRJNA721257), and the root (RT), stem (SM), mature leaf (ML), fruit (FT) and leaf bud (LB) were obtained from our previous study (GEO number: GSE142491). The FPKM was used to estimate the significance among three *ZaMBF*1 genes in each sample by one-way ANOVA test, and the level of significance was set to *p* < 0.05. Meanwhile, eight samples were used to uncover the expression patterns of *ZaMBF1* during male and female floral differentiation and development processes, including male stage 1 (M1), male stage 2 (M2), male stage 3 (M3), male stage 4 (M4), female stage 1 (F1), female stage 2 (F2), female stage 3 (F3), and female stage 4 (F4) (GEO number: GSE195749), and the significance of gene expression was calculated by DEseq2 software [79], all of the up- or down-regulation are illustrated by the first comparison component. Additionally, the six transcriptome profiles were also analysed after infecting the plant with leaf rust disease from our recent studies, including INCZ0, INCZ1, INCZ3, INCZ7, INCZ14, INCZ21. All of the transcriptome data were collected from three biological replicates. The expression of *ZaMBF1* were displayed as heatmap by TBtools software [74].

### Exogenous treatments and quantitative real-time PCR (qRT-PCR) analysis

One-year-old seedlings of *Z. armatum* were planted in a plastic pot as the experimental material in this study, which was located in Sichuan Agricultural University (30.70°N, 103.87°E), Chengdu City, Sichuan Province, China. The seedlings were treated with the different abiotic stress and phytohormone inductions, including water-logging, cold stress, ethephon, methyl jasmonic acid (MeJA), and salicylic acid (SA). Each treatment had at least three biological repeats. Because the seedlings died within approximately one week under flood conditions, similarly grown seedlings of *Z. armatum* (50–60 cm high with 35–45 leaf nodes) were selected for enough irrigation with water to submerge the soil for 0, 6 h, 12 h, 1 d, 2d, 3d, and 6 d as the water-logging treatment in this study. Meanwhile, the 5–6 °C is the lowest temperature for the survival of *Z. armatum* in the wild, so similarly grown seedlings were grown at 6 °C for 0, 0.5, 1, 2, 3, 6, 9, 12, and 24 h as the cold stress treatment. For phytohormone analysis, similarly grown seedlings of *Z. armatum* were treated with 2.5% v/v ethephon (40% ingredient), 100 M MeJA or 100 M SA (Sigma, Santa Clara, CA, USA) solution for 0, 3, 6, 9, 12, and 24 h as described in previous studies [1, 42]. At selected time points of various treatments, similarly sized leaves were collected and flash frozen in liquid nitrogen, and deposited in an ultra-low temperature freezer (Thermo, Waltham, MA, USA).

The total RNA of each sample was extracted using a FastPure Plant Total RNA Isolation Kit (Vazyme, Nanjing, China) according to the manufacturer’s protocol, and cDNA was synthesized using a PrimeScript® II First Strand cDNA Synthesis Kit (TaKaRa, Japan). The specific primers were obtained by Primer Premier 5.0 with amplified PCR products from 80 to 300 bp (Additional file 2: Table S2). The qRT-PCR was performed according to our previous experimental procedure [24, 78]. The 0 h of each treatment was used as a control sample to estimate the relative expression of *ZaMBF1* in this study. All of the data was arranged and calculated using Excel 2019 software. The analysis of variance was performed using R version 4.0.3 software, and the level of significance was set to *p* < 0.05. The GraphPad Prism 5 was used to draw the Figures [80].

## Supplementary Information

**Additional file 1:** **Supplementary material 1. Table S1. **The information of MBF1 family detected in different plants.

**Additional file 2:** **Supplementary material 2. Table S2. **The ZaMBF1 primers of qRT-PCR used for detecting the expression patterns in *Zanthoxylum armatum.*

**Additional file 3:** **Supplementary material 3. Table S3. **The summary of the MBF1s annotation against in Pfam database.

**Additional file 4:** **Supplementary material 4. Table S4. **The motifs information discovered in MBF1 protein sequences.

**Additional file 5:** **Supplementary material 5. Table S5. **The domain information discovered in MBF1 protein sequences.

**Additional file 6:** **Supplementary material 6. Figure S1. **The chromosomal locations of *MBF1* genes from six representative species.At is *A. thaliana*, Os is *O. sativa*, Pt is *P. trichocarpa*, Cs is *C. sinensis*, Gb is *G. biloba.*

**Additional file 7:** **Supplementary material 7. Table S6. **The detailed information of cis-elements detected in the MBF1 family of various species.

**Additional file 8:** **Supplementary material 8. Table S7. **The number of functional element regions detected in MBF1 promoter of various speices.

**Additional file 9:** **Supplementary material 9. Table S8. **The number of functional element regions detected in MBF1 promoter of various speices.

**Additional file 10:** **Supplementary material 10. Table S9. **The information associated with the target gene analysis of ZaMBF1 family.

**Additional file 11:** **Supplementary material 11. Table S10. **The expression abundance of ZaMBF1 family in various tissues. The Genome means the data was analyzed using the public data in *Z. armatum*, the Transcriptome means the data was obtained from our previous transcriptome database. The detailed information was illustruated in Material and methods of our manuscript.

**Additional file 12:** **Supplementary material 12. Table S11. **The expression of *ZaMBF1* family in various tissues.

## Data Availability

The data supporting the results presented in this article are included as additional files.
