# Supplementary figures and images for: Genome-wide characterization of the MBF1 gene family and its expression pattern in different tissues and stresses in Zanthoxylum armatum
Source: BMC Genomics. 2022 Sep 14;23:652. doi: 10.1186/s12864-022-08863-4 (PMC9472409; doi:10.1186/s12864-022-08863-4)

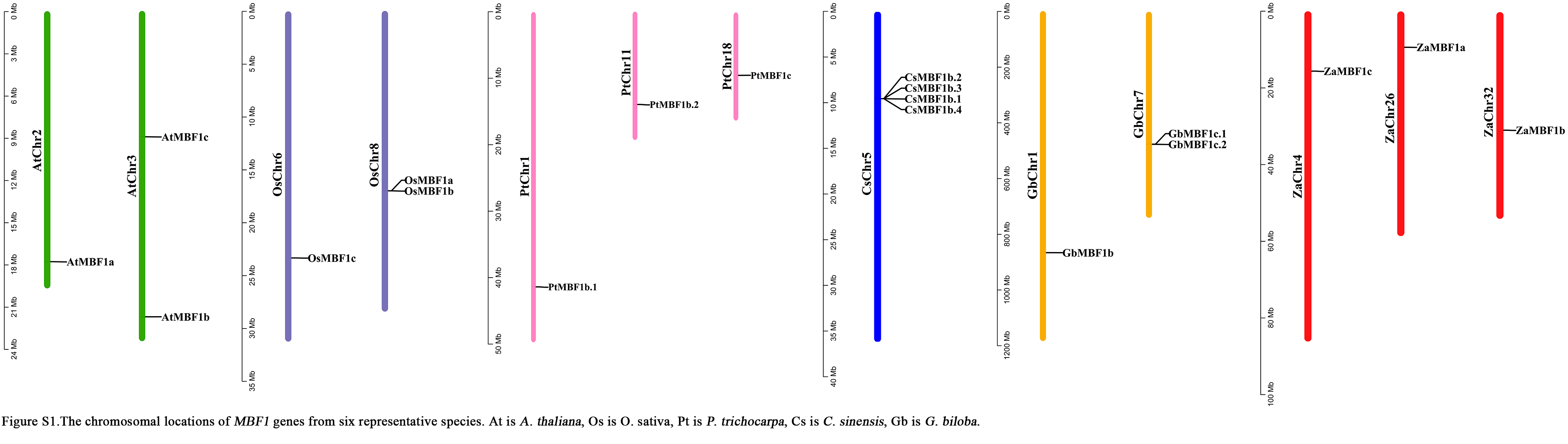

Supplement: Supplementary file 6 — Additional file 6: Supplementary material 6. Figure S1. The chromosomal locations of MBF1 genes from six representative species.At is A. thaliana, Os is O. sativa, Pt is P. trichocarpa, Cs is C. sinensis, Gb is G. biloba. [file 12864_2022_8863_MOESM6_ESM.jpg]
